# Association of dietary diversity measured by the number of dishes with cardiovascular risk factors among Japanese adults: findings from the National Health and Nutrition Survey, 2018-19

**DOI:** 10.1186/s12937-026-01326-6

**Published:** 2026-05-12

**Authors:** Saeka Takabayashi, Emiko Okada, Hidemi Takimoto, Mieko Nakamura, Satoshi Sasaki, Kunihiko Takahashi, Koshi Nakamura, Shigekazu Ukawa, Akiko Tamakoshi

**Affiliations:** 1 Department of Public Health, Graduate School of Medicine, Hokkaido University, North 15 West 7 Kita-ku, Sapporo, 060-8638 Japan; 2 The Health Care Science Institute, 3-2-12 Akasaka, Minato-ku, Tokyo, 107-0052 Japan; 3 National Institutes of Biomedical Innovation, Health and Nutrition, Kento Innovation Park, NK Building, 3-17 Senrioka Shinmachi, Settsu-shi, Osaka 566-0002 Japan; 4 Center for Nutritional Epidemiology and Policy Research, National Institutes of Biomedical Innovation, Health and Nutrition, Kento Innovation Park, NK Building, 3-17 Senrioka Shinmachi, Settsu-shi, Osaka 566-0002 Japan; 5 Department of Social and Preventive Epidemiology, School of Public Health, Graduate School of Medicine, The University of Tokyo, Hongo 7-3-1, Bunkyo-ku, Tokyo, 113-0033 Japan; 6 Department of Biostatistics, M&D Data Science Center, Institute of Integrated Research, Institute of Science Tokyo, Tokyo, 113-8510 Japan; 7 Department of Public Health and Epidemiology, Graduate School of Medicine, University of the Ryukyus, Ginowan, 901-2720 Japan; 8 Graduate School of Human Life and Ecology, Osaka Metropolitan University, Sugimoto 3-3-138, Sumiyoshi-ku, Osaka, 558-8585 Japan; 9 Department of Public Health, Hokkaido University Faculty of Medicine, North 15 West 7 Kita-ku, Sapporo, 060-8638 Japan

**Keywords:** Number of dishes, Dietary diversity, Cardiovascular disease

## Abstract

**Background:**

The Japanese-style diet has attracted attention as a factor contributing to the Japanese population’s longevity by reducing cardiovascular disease (CVD) mortality. The Japanese-style diet is said to be "ichijusansai" (rice with one soup and three dishes) and is characterized by a high number of dishes. However, the relationship between the number of dishes in all meals (NDAM) and CVD risk factors remains unclear.

**Methods:**

In this cross-sectional study, we analyzed data from 2,900 participants aged ≥20 years from the 2018-19 National Health and Nutrition Survey. Participants were divided into four groups according to sex and NDAM. NDAM was calculated based on a one-day weighed dietary record, excluding beverages, and including all other dishes and foods. Multivariable relative risks (RRs) were estimated using a modified Poisson regression model adjusted for age, living alone, area, occupation, exercise habits, smoking habits, drinking habits, and total energy intake.

**Results:**

Participants with higher NDAM were more likely to be older, non-smokers, and physically active. In men, higher NDAM (Groups 2–4) was associated with a lower risk of dyslipidemia compared to Group 1 (the lowest NDAM). In women, an inverse association was observed for overweight/obesity, with Group 4 (the highest NDAM) showing a lower risk than Group 1. Furthermore, a lower risk of hypertension was found in Groups 2–3 compared to Group 1. However, these associations did not remain statistically significant after Bonferroni correction.

**Conclusion:**

A diet with a high NDAM may be inversely associated with several CVD risk factors among Japanese adults.

**Supplementary Information:**

The online version contains supplementary material available at 10.1186/s12937-026-01326-6.

## Background

According to Organization for Economic Co-operation and Development (OECD) data (2021), Japan has the highest average life expectancy in the world, with men living an average of 80.5 years, women 87.7 years, and an overall average of 84.7 years. One of the factors contributing to Japan’s remarkable longevity is the Japanese-style diet [1], which has garnered significant attention. This diet is primarily characterized by the frequent consumption of rice, soy products, fish, and green tea, all of which are associated with lower rates of cardiovascular disease (CVD) and overall mortality [2, 3]. CVD is a leading cause of premature death and morbidity worldwide, with known risk factors including obesity, hypertension, diabetes, and dyslipidemia [4].

Although the Japanese-style diet is defined by specific foods and food groups, it is also recognized for its wide variety of dishes. This variety is exemplified by the traditional meal format known as *ichijusansai* (rice with one soup and three dishes), which typically consists of a dietary staple, a main dish, two side dishes, and a soup. A previous study evaluating a Japanese-style diet based on the number of dishes in all meals a day (NDAM) found that NDAM was associated with nutritional adequacy to the same extent as widely used dietary diversity indices, such as the food variety score (number of food items) and dietary diversity score (number of food groups) [5]. However, the study also revealed that NDAM was associated with nutritional characteristics, such as high sodium and dietary cholesterol intake, that could increase the risk of CVD [5]. High sodium intake is a well-established risk factor for hypertension [6], while high dietary cholesterol intake is a known risk factor for dyslipidemia [7].

Although greater adherence to a Japanese-style diet, when evaluated by food intake patterns, has been reported to be associated with lower blood pressure in both men and women [8] and lower serum low density lipoprotein (LDL) cholesterol in men [9], these previous studies focused mainly on dietary patterns or food groups rather than a dish-based assessment. Evidence on the association between NDAM, a dish-based dietary diversity indicator that reflects one aspect of the Japanese-style diet, and CVD risk factors remains limited. Therefore, this study aimed to investigate whether NDAM is associated with CVD risk factors such as overweight/obesity, abdominal obesity, hypertension, diabetes, and dyslipidemia.

## Methods

### Study design and study population

Data were collected from the National Health and Nutrition Survey (NHNS) in 2018–19, a cross-sectional survey conducted annually every November by the Ministry of Health, Labor, and Welfare in Japan since 1947. Details of the survey design have been reported elsewhere [10]. Briefly, the NHNS surveys approximately 300 areas each year, which are selected using stratified random sampling. The survey comprises the following three components: (1) physical examinations of household members, (2) household-based dietary surveys, and (3) a questionnaire survey on dietary habits and lifestyles. Of the 9,497 eligible households, 6,104 (64.3%) participated in the dietary survey. The sample was considered representative of the Japanese population. Of the 12,459 participants aged ≥ 20 years, exclusions were applied to those not participating in dietary surveys (*n* = 1,789), pregnant or lactating women (*n* = 135), individuals without blood test data (*n* = 5,345), those currently on medication (anti-hypertensive, anti-hyperglycemic, cholesterol-lowering, or serum triglyceride-lowering) or diagnosed with diabetes mellitus (*n* = 2,268), and those for whom body mass index (BMI), waist circumference (WC), blood pressure, diabetes mellitus, or hyperlipidemia (*n* = 22) could not be measured. The final analysis included 2,900 participants. Our study was exempt from the Ethical Guidelines for Epidemiological Research published by the Ministry of Health, Labor, and Welfare, as it involved the secondary use of preexisting, anonymized data.

### Dietary assessment

The NHNS dietary surveys in 2018 and 2019 were conducted using a one-day weighed dietary record. Trained dietitians visited each household and instructed the individual primarily responsible for meal preparation to complete a dietary record. Participants were asked to record all cooked dishes, their ingredients, the total amount prepared, any leftovers, and the approximate proportion of each food item consumed by each family member. The type and amount of food consumed outside the home were also recorded for each individual. Dietitians subsequently reviewed all submitted records for completeness.

### Calculation of individual daily intake of foods, energy, nutrients, and NDAM

Individual food consumption was calculated on the basis of the household dietary surveys using the Standard Tables of Food Composition in Japan (2015 edition) [11]. Specifically, the amount of food consumed by each individual was determined from the household-based food-weight records and the reported proportions consumed by each family member. The validity of estimating individual energy and nutrient intake from this household-based dietary record method has been previously established [12]. Intake of food groups and nutrients was energy-adjusted using the density method (i.e., amount/1000 kcal) [13].

Dishes were recorded for each meal, including breakfast, lunch, dinner, and snacks. Details of the NDAM calculation have been reported in a previous study [5]. Briefly, a dish was counted toward an individual’s NDAM if its consumption exceeded 0 g. All food items, including grain dishes, main dishes, side dishes, milk, and fruits were included in the calculation, except for beverages. If the same dish (e.g., rice) was consumed several times a day, it was counted only once.

### Calculation of dietary energy density

The dietary energy density was calculated by dividing the reported energy intake (kcal/day) by the reported weight of all foods consumed (g/day). This approach is consistent with that used in previous studies [14, 15]. This calculation is based on solid foods only; all caloric and non-caloric beverages (e.g., water, fruit and vegetable juice, milk, tea, coffee, soft drinks, and alcoholic beverages) were excluded.

### Anthropometric and clinical measures

Anthropometric measurements, including height, weight, and WC, were obtained using NHNS protocols. Clinical and biochemical measurements, including blood pressure, hemoglobin A1c (HbA1c), total cholesterol, HDL cholesterol (HDL-C) and LDL cholesterol (LDL-C), and triglyceride (TG), were also obtained. BMI was calculated as body weight (kg) divided by height (m) squared.

### Definitions of clinical conditions

Overweight/obesity and abdominal obesity were assessed separately because BMI reflects overall adiposity, whereas waist circumference reflects central adiposity. Overweight/obesity was defined as BMI ≥ 25 kg/m^2^ and abdominal obesity was defined as WC ≥ 85 cm for men and WC ≥ 90 cm for women. Hypertension was defined as a systolic blood pressure ≥ 140 mmHg or a diastolic blood pressure ≥ 90 mmHg. Diabetes mellitus was defined as HbA1c ≥ 6.5%. Dyslipidemia was defined as meeting one or more of the following criteria: LDL-C ≥ 120 mg/dL, HDL-C < 40 mg/dL, non-HDL cholesterol (non-HDL-C; calculated as total cholesterol – HDL-C) ≥ 150 mg/dL, and/or TG ≥ 150 mg/dL.

### Statistical analyses

**Fig. 1:**
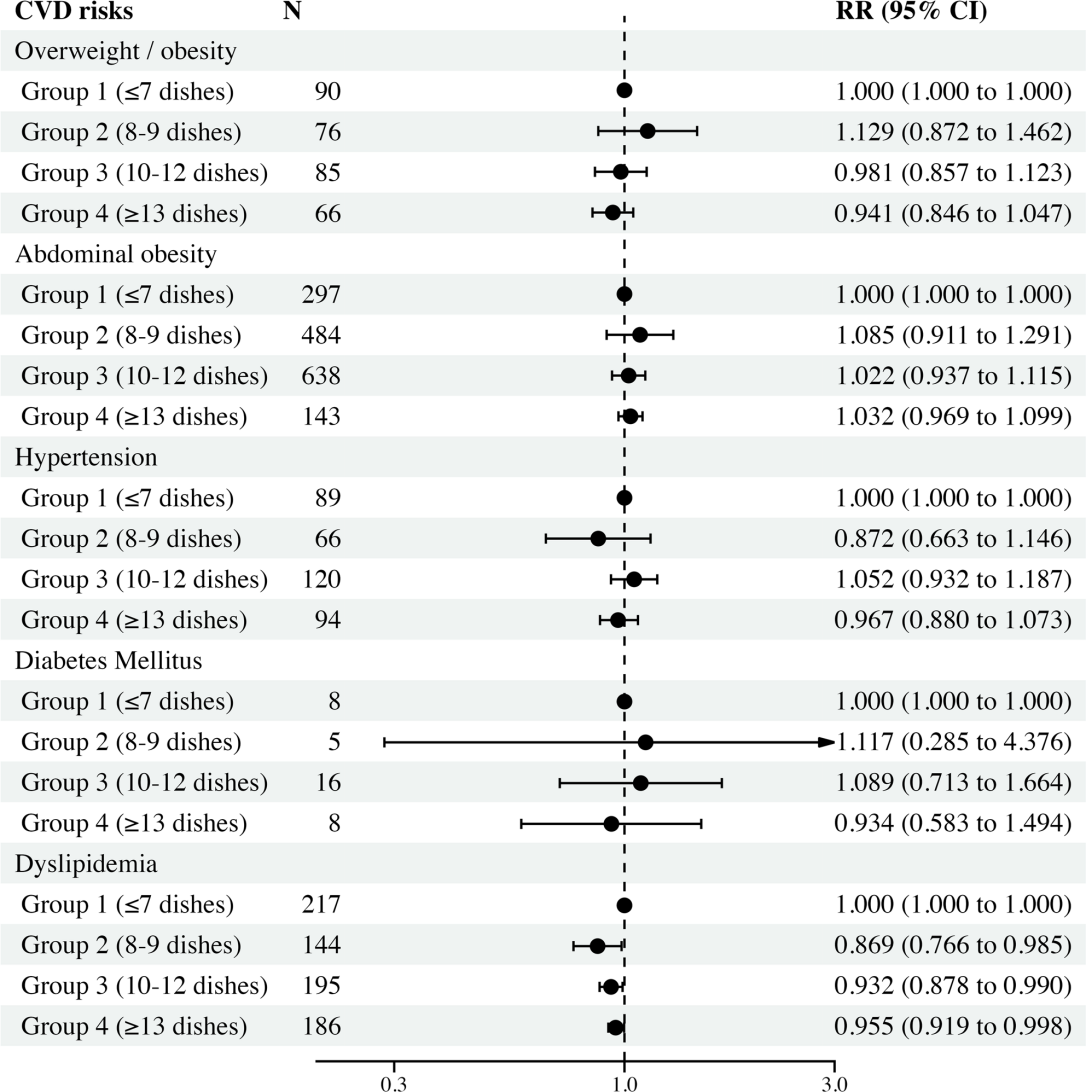
Multivariable-adjusted RRs and 95% CIs for CVD risk factors according to the NDAM in men. RR: Risk ratios, CI: confidence intervals, CVD: cardiovascular disease, NDAM: number of dishes in all meals. Analyzed by modified Poisson regression model. Multivariable model: adjusted for age, living alone, area, occupation, smoking status, drinking status, exercise habits, and total energy intake. Note: Error bars indicate 95% confidence intervals (CIs). Statistical significance was assessed using the Bonferroni correction

**Fig. 2:**
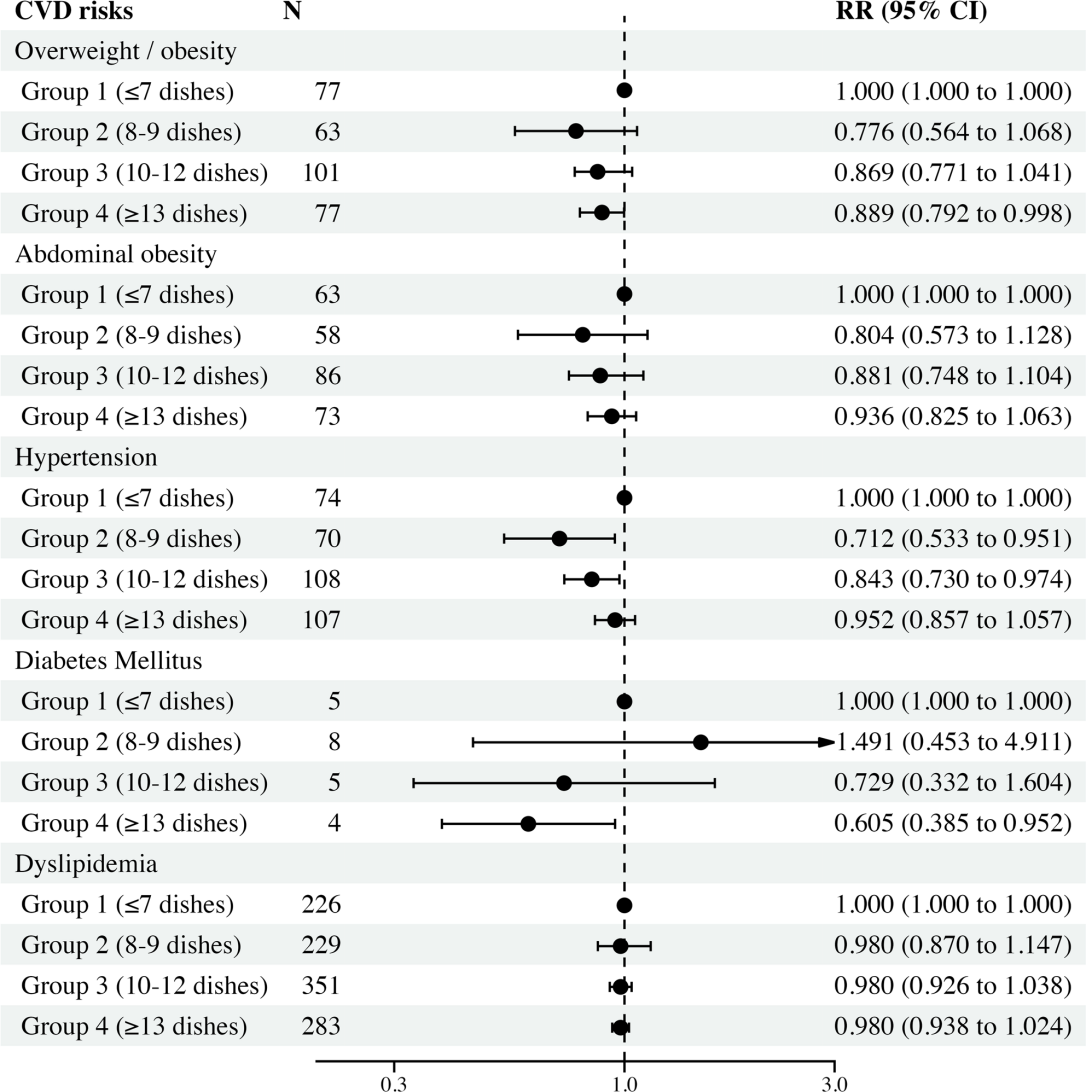
Multivariable-adjusted RRs and 95% CIs for CVD risk factors according to the NDAM in women. RR: Risk ratios, CI: confidence intervals, CVD: cardiovascular disease, NDAM: number of dishes in all meals. Analyzed by modified Poisson regression model. Multivariable model: adjusted for age, living alone, area, occupation, smoking status, drinking status, exercise habits, and total energy intake. Note: Error bars indicate 95% confidence intervals (CIs). Statistical significance was assessed using the Bonferroni correction

The analyses were stratified according to sex. For each sex, participants were divided into four groups based on their NDAM score: Group 1 (≤ 7 dishes), Group 2 (8–9 dishes), Group 3 (10–12 dishes), and Group 4 (≥ 13 dishes). The participant characteristics are presented for each group (Tables 1 and 2). To examine the association between NDAM and CVD risk factors, multivariable relative risks (RRs) were estimated using modified Poisson regression models with robust error variance [16]. The models were adjusted for the following variables obtained from the questionnaires: age groups (20–29, 30–39, 40 − 49, 50 − 59, 60 − 69, 70–79, or ≥ 80 years), living alone (yes or no), area (Hokkaido, Tohoku, Kanto Ⅰ, Kanto Ⅱ, Hokuriku, Tokai, Kinki I, Kinki II, Chugoku, Shikoku, Kitakyushu, or Minamikyushu), occupation (professional/technical work, managerial, clerical, sales, services, security services, agriculture, forestry, fisheries, transportation/communications, manufacturing, housekeepers/domestic helper, student, other, or unknown), smoking status (current, former, never, or unknown), drinking status (current, non-current, or unknown), exercise habits (yes, no, or unknown), and total energy intake. Current drinkers were defined as participants who consumed ≥ 1 sake equivalent on three or more days per week. The exercise habit was considered present (“yes”) if a participant engaged in at least 30 min of physical activity on two or more days per week, sustained for at least one year. The *P* for the trend was calculated across the median NDAM values in each group. Two analytical approaches were used to address potential confounding effects of medication. In the primary analysis, all individuals taking the relevant medications were excluded. In the sensitivity analysis, only individuals taking medications specific to the outcome under study were excluded to maximize the sample size. This approach meant that no participants were excluded from the analyses of overweight/obesity and abdominal obesity, whereas analyses for other conditions involved targeted exclusions. Accordingly, the hypertension analysis was limited to individuals not taking anti-hypertensive medication, the diabetes analysis to those not taking anti-hyperglycemic drugs, and the dyslipidemia analysis to those not taking lipid-lowering agents. The threshold for statistical significance was set at a two-sided P-value of < 0.05. However, for the primary outcomes presented in Figs. 1 and 2, the statistical significance level was adjusted using the Bonferroni method to account for multiple comparisons (α = 0.05/20 = 0.0025). This correction factor of 20 was derived from the fact that four tests (three pairwise comparisons of Groups 2, 3, and 4 against Group 1, plus a test for trend) were performed for the five outcomes. Confidence intervals (CIs) are presented at the nominal 95% level to indicate the precision of the point estimates and were not adjusted for multiple comparisons. All statistical analyses were performed using JMP Pro 17 for Mac (SAS Institute, Cary, NC, USA) or R (version 4.2.2).

**Table 1:** Characteristics of participants according to NDAM in men

	Men (*n* = 1,100)			
	≤ 7 dishes (*n* = 305, 27.7%)	8–9 dishes (*n* = 221, 20.0%)	10–12 dishes (*n* = 304, 27.6%)	≥ 13 dishes (*n* = 271, 24.6%)
Age (years) (n, %)				
20–29	39 (12.7)	20 (9.1)	24 (7.9)	15 (5.5)
30–39	61 (20.0)	40 (18.2)	31 (10.2)	21 (7.7)
40–49	77 (25.3)	34 (15.4)	47 (15.5)	42 (15.4)
50–59	41 (13.4)	38 (17.2)	57 (18.8)	29 (10.7)
60–69	41 (13.4)	41 (18.6)	59 (19.4)	77 (28.3)
70–79	37 (12.1)	32 (14.5)	71 (23.4)	68 (25.1)
≥80	9 (2.9)	15 (6.8)	15 (4.9)	19 (7.0)
Living alone (n, %)				
Yes	66 (21.6)	24 (10.9)	34 (11.2)	24 (8.9)
No	239 (78.4)	196 (89.1)	270 (88.8)	247 (91.1)
Area				
Hokkaido	8 (2.6)	10 (4.5)	11 (3.6)	8 (3.0)
Tohoku	22 (7.2)	21 (9.6)	30 (9.9)	16 (5.9)
KantoⅠ	48 (15.7)	31 (14.1)	47 (15.5)	59 (21.7)
Kanto II	30 (9.8)	17 (7.7)	31 (10.2)	26 (9.6)
Hokuriku	14 (4.6)	11 (5.0)	14 (4.6)	14 (5.2)
Tokai	45 (14.8)	28 (12.7)	33 (10.9)	43 (15.9)
Kinki Ⅰ	37 (12.1)	24 (10.9)	31 (10.2)	21 (7.8)
Kinki Ⅱ	20 (6.8)	6 (2.7)	16 (5.3)	13 (4.8)
Chugoku	20 (6.6)	20 (9.1)	34 (11.2)	20 (7.4)
Shikoku	9 (3.0)	12 (5.5)	17 (5.6)	20 (7.4)
Kita (Northern) Kyushu	29 (9.5)	24 (10.9)	19 (6.3)	17 (6.3)
Minami (Southern) Kyushu	22 (7.2)	16 (7.3)	21 (6.9)	14 (5.2)
Occupation (n, %)				
Professional/Technical Work	37 (12.1)	32 (14.6)	43 (14.1)	32 (11.8)
Managerial	24 (7.9)	14 (6.4)	18 (5.9)	20 (7.4)
Clerks	27 (8.9)	17 (7.7)	23 (7.6)	28 (10.3)
Sales	23 (7.5)	20 (9.1)	16 (5.3)	12 (4.4)
Services	20 (6.6)	18 (8.2)	22 (7.2)	14 (5.2)
Security services	11 (3.6)	7 (3.2)	9 (3.0)	3 (1.1)
Agriculture	16 (5.3)	20 (9.1)	24 (7.9)	20 (7.4)
Forestry	0 (0.0)	2 (0.9)	1 (0.3)	0 (0.0)
Fisheries	1 (0.3)	0 (0.0)	0 (0.0)	1 (0.4)
Transportation/Communications	14 (4.6)	10 (4.6)	11 (3.6)	5 (1.9)
Manufacturing	64 (21.0)	38 (17.3)	44 (14.5)	46 (17.0)
Housekeepers/Domestic Helpers	6 (2.0)	7 (3.2)	8 (2.6)	10 (3.7)
Students	10 (3.3)	4 (1.8)	4 (1.3)	3 (1.1)
Other	52 (17.1)	31 (14.1)	81 (26.6)	77 (28.4)
Smoking status (n, %)				
Current smoker	113 (37.1)	67 (30.5)	87 (28.6)	52 (19.2)
Past smoker	33 (10.8)	28 (12.7)	38 (12.5)	41 (15.1)
Non-smoker	158 (51.8)	125 (56.8)	179 (58.9)	177 (65.3)
Unknown	1 (0.3)	0 (0.0)	0 (0.0)	1 (0.4)
Drinking status (n, %)				
Current drinker	91 (29.8)	75 (34.1)	105 (34.5)	80 (29.5)
Non-current drinker	213 (69.8)	145 (65.9)	199 (65.5)	191 (70.1)
Unknown	1 (0.3)	0 (0.0)	0 (0.0)	1 (0.4)
Habitual exercise (n, %)				
Yes	73 (23.9)	64 (29.2)	94 (30.9)	95 (35.1)
No	227 (74.4)	150 (68.5)	203 (66.8)	171 (62.7)
Unknown	5 (1.6)	5 (2.3)	7 (2.3)	6 (2.2)
Food groups intake (mean, SD)				
Cereals, g/1000 kcal	245 (89)	238 (73)	226 (655)	202 (59)
Potatoes, g/1000 kcal	21 (31)	22 (31)	21 (27)	26 (29)
Sugars and Sweeteners, g/1000 kcal	3 (5)	4 (4)	3 (4)	3 (4)
Pulses, g/1000 kcal	25 (38)	27 (33)	29 (31)	34 (32)
Vegetables, g/1000 kcal	110 (83)	118 (74)	136 (79)	145 (80)
Fruits, g/1000 kcal	17 (48)	30 (48)	41 (55)	67 (60)
Mushrooms, g/1000 kcal	6 (14)	7 (12)	9 (15)	10 (15)
Algae, g/1000 kcal	3 (7)	4 (6)	5 (8)	6 (9)
Fishes and Shellfish, g/1000 kcal	25 (33)	35 (36)	37 (32)	37 (29)
Meats, g/1000 kcal	67 (52)	55 (38)	53 (35)	49 (31)
Eggs, g/1000 kcal	20 (22)	19 (19)	21 (18)	22 (16)
Milk, g/1000 kcal	32 (62)	44 (62)	50 (65)	71 (621)
Fats and Oils, g/1000 kcal	6 (5)	6 (5)	6 (4)	6 (4)
Confectioneries, g/1000 kcal	9 (26)	9 (18)	11 (19)	14 (19)
Nutrient intake (mean, SD)				
Total energy, kcal/d	2,001 (626)	2,222 (554)	2,355 (540)	2,507 (557)
Energy Density, kcal/g	1.83 (0.50)	1.72 (0.37)	1.65 (0.35)	1.59 (0.32)
Protein, % energy	14.17 (3.58)	14.36 (3.07)	14.56 (2.98)	14.83 (2.63)
Animal protein, % energy	7.96 (3.93)	7.92 (3.31)	8.16 (3.10)	8.20 (2.69)
Plant protein, % energy	6.22 (1.87)	6.45 (1.63)	6.41 (1.57)	6.63 (1.54)
Fat, % energy	27.46 (8.84)	27.23 (6.69)	27.32 (7.33)	28.01 (6.48)
Monounsaturated fatty acids, % energy	10.49 (3.94)	10.07 (3.01)	10.26 (3.44)	10.34 (2.88)
Polyunsaturated fatty acids, % energy	5.79 (2.58)	5.95 (1.82)	6.03 (1.85)	6.22 (1.97)
n-3 fatty acids, % energy	0.98 (0.75)	1.10 (0.64)	1.19 (0.67)	1.17 (0.65)
n-6 fatty acids, % energy	4.77 (2.19)	4.80 (1.55)	4.79 (1.52)	5.02 (1.62)
Saturated Fat, % energy	8.01 (3.27)	7.92 (2.72)	7.66 (2.85)	8.00 (2.44)
Carbohydrate, % energy	51.93 (11.02)	52.48 (8.74)	52.28 (9.23)	52.74 (8.34)
Dietary Fiber, g/1000 kcal	6.83 (2.99)	7.58 (2.62)	8.04 (2.95)	9.04 (3.33)
Magnesium, mg/1000 kcal	117 (46)	125 (36)	131 (40)	143 (40)
Sodium, mg/1000 kcal	1,976 (724)	2,052 (630)	1,937 (575)	1,985 (576)
Potassium, mg/1000 kcal	978 (332)	1,064 (290)	1,134 (325)	1,294 (367)
Sodium-to-Potassium Ratio	2.16 (0.91)	2.03 (0.73)	1.81 (0.64)	1.62 (0.53)
Cholesterol, mg/1000 kcal	162 0.8 (97.5)	161.2 (88 0.9)	171.4 (88.3)	178.4 (76.5)

*NDAM* Number of dishes in all meals

**Table 2:** Characteristics of participants according to NDAM in women

	Women (*n* = 1,800)			
	≤ 7 dishes (*n* = 393, 21. %)	8–9 dishes (*n* = 386, 21.4%)	10–12 dishes (*n* = 567, 31.5%)	≥ 13 dishes (*n* = 454, 25.2%)
Age (years) (n, %)				
20–29	39 (9.9)	19 (4.9)	30 (5.3)	15 (3.3)
30–39	74 (18.8)	65 (16.8)	54 (9.5)	35 (7.7)
40–49	108 (27.5)	90 (23.3)	118 (20.8)	72 (15.9)
50–59	87 (22.1)	74 (19.2)	119 (21.0)	91 (20.0)
60–69	42 (10.7)	75 (19.4)	133 (23.5)	138 (30.4)
70–79	30 (7.6)	44 (11.4)	83 (14.6)	79 (17.4)
≥80	13 (3.3)	19 (4.9)	30 (5.3)	24 (5.3)
Living alone (n, %)				
Yes	46 (11.7)	40 (10.4)	67 (11.8)	46 (10.1)
No	347 (88.3)	346 (89.6)	500 (88.2)	408 (89.9)
Area				
Hokkaido	13 (3.3)	16 (4.2)	10 (1.8)	14 (3.1)
Tohoku	25 (6.4)	24 (6.2)	53 (9.4)	53 (11.7)
KantoⅠ	69 (17.6)	63 (16.3)	107 (18.9)	94 (20.7)
Kanto II	23 (5.9)	39 (10.1)	44 (7.8)	38 (8.4)
Hokuriku	22 (5.6)	23 (6.0)	44 (7.8)	28 (6.2)
Tokai	42 (10.7)	46 (11.9)	74 (13.1)	64 (14.1)
Kinki Ⅰ	69 (17.6)	51 (13.2)	63 (11.1)	47 (10.4)
Kinki Ⅱ	18 (4.6)	13 (3.4)	19 (3.4)	18 (4.0)
Chugoku	30 (7.6)	38 (9.8)	40 (7.1)	22 (4.9)
Shikoku	20 (5.1)	18 (4.7)	30 (5.3)	20 (4.4)
Kita (Northern) Kyushu	33 (8.4)	33 (8.6)	40 (7.1)	33 (7.3)
Minami (Southern) Kyushu	29(7.4)	22 (5.7)	43 (7.6)	23 (5.1)
Occupation (n, %)				
Professional/Technical Work	58 (14.8)	65 (16.8)	107 (18.9)	54 (11.9)
Managerial	2 (0.5)	0 (0.0)	2 (0.4)	2 (0.4)
Clerks	59 (15.0)	52 (13.5)	78 (13.8)	68 (15.0)
Sales	29 (7.4)	15 (3.9)	18 (3.2)	14 (3.1)
Services	78 (19.9)	59 (15.3)	61 (10.8)	57 (12.6)
Security services	0 (0.0)	0 (0.0)	1 (0.2)	0 (0.0)
Agriculture	5 (1.3)	7 (1.8)	25 (4.4)	15 (3.3)
Forestry	0 (0.0)	1 (0.3)	1 (0.2)	0 (0.0)
Fisheries	0 (0.0)	0 (0.0)	0 (0.0)	0 (0.0)
Transportation/Communications	0 (0.0)	1 (0.3)	1 (0.2)	0 (0.0)
Manufacturing	25 (6.4)	26 (6.7)	31 (5.5)	23 (5.1)
Housekeepers/Domestic Helpers	115 (29.3)	133 (34.4)	202 (35.6)	189 (41.6)
Students	4 (1.0)	5 (1.3)	8 (1.4)	0 (0.0)
Other	18(4.6)	23 (6.0)	33 (5.8)	32 (7.1)
Smoking status (n, %)				
Current smoker	60 (15.3)	35 (9.1)	34 (6.0)	15 (3.3)
Past smoker	24 (6.1)	13 (3.4)	22 (3.9)	8 (1.8)
Non-smoker	v (78.4)	337 (87.3)	508 (89.6)	431 (94.9)
Unknown	1 (0.3)	1 (0.3)	3 (0.5)	0 (0.0)
Drinking status (n, %)				
Current drinker	52 (13.2)	42 (10.9)	47 (8.3)	26 (5.7)
Non-current drinker	340 (86.5)	342 (88.8)	517 (91.2)	428 (94.3)
Unknown	1 (0.3)	1 (0.3)	3(0.5)	0 (0.0)
Habitual exercise (n, %)				
Yes	67 (17.1)	74 (19.2)	116 (20.5)	133 (29.4)
No	318 (80.9)	303 (78.7)	445 (78.5)	313 (69.3)
Unknown	8 (2.0)	8 (2.1)	6 (1.1)	6 (1.3)
Food groups intake (mean, SD)				
Cereals, g/1000 kcal	236 (86)	215 (75)	201 (63)	179 (55)
Potatoes, g/1000 kcal	26 (41)	26 (34)	31 (37)	28 (32)
Sugars and Sweeteners, g/1000 kcal	4 (5)	4 (4)	4 (5)	4 (4)
Pulses, g/1000 kcal	31 (54)	32 (45)	39 (46)	41 (45)
Vegetables, g/1000 kcal	136 (83)	151 (93)	165 (89)	169 (92)
Fruits, g/1000 kcal	33 (64)	57 (78)	62 (63)	83 (62)
Mushrooms, g/1000 kcal	9 (17)	10 (19)	10 (15)	11 (15)
Algae, g/1000 kcal	4 (10)	5 (11)	5 (11)	6 (10)
Fishes and Shellfish, g/1000 kcal	26 (36)	33 (34)	35 (32)	36 (31)
Meats, g/1000 kcal	58 (45)	55 (37)	52 (37)	45 (29)
Eggs, g/1000 kcal	21 (25)	23 (22)	23 (19)	25 (17)
Milk, g/1000 kcal	50(76)	62 (75)	72 (72)	92 (77)
Fats and Oils, g/1000 kcal	6 (5)	6 (5)	6 (5)	6 (5)
Confectioneries, g/1000 kcal	13(29)	16(26)	16 (24)	20 (23)
Nutrient intake (mean, SD)				
Total energy, kcal/d	1,480 (390)	1,697 (417)	1,810 (410)	2,025 (493)
Energy Density, kcal/g	1.64 (0.43)	1.57 (0.37)	1.50 (0.32)	1.48 (0.32)
Protein, % energy	14.68 (3.38)	15.13 (2.96)	15.49 (3.10)	15.59 (2.91)
Animal protein, % energy	7.75 (3.62)	8.23 (3.02)	8.39 (3.10)	8.49 (3.0)
Plant protein, % energy	6.95 (1.88)	6.91 (1.65)	7.11 (1.66)	7.11 (1.48)
Fat, % energy	28.50 (8.32)	29.41 (7.94)	29.20 (7.37)	29.73 (6.31)
Monounsaturated fatty acids, % energy	10.49 (3.67)	10.78 (3.60)	10.58 (3.35)	10.68 (2.90)
Polyunsaturated fatty acids, % energy	5.88 (2.28)	6.24 (2.26)	6.43 (2.22)	6.55 (2.06)
n-3 fatty acids, % energy	0.98 (0.68)	1.08 (0.66)	1.17 (0.73)	1.25 (0.72)
n-6 fatty acids, % energy	4.85 (1.96)	5.10 (1.96)	5.23 (1.88)	5.26 (1.72)
Saturated Fat, % energy	8.63 (3.43)	8.81 (3.21)	8.59 (2.83)	8.73 (2.48)
Carbohydrate, % energy	53.29 (9.92)	52.97 (9.19)	52.93 (8.73)	53.26 (7.30)
Dietary Fiber, g/1000 kcal	8.26 (3.08)	9.02 (3.27)	9.71 (3.30)	10.02 (3.3)
Magnesium, mg/1000 kcal	131 (47)	140 (43)	150 (44)	156 (45)
Sodium, mg/1000 kcal	2,202 (768)	2,139 (700)	2,121 (661)	2,096 (657)
Potassium, mg/1000 kcal	1,143(376)	1,266 (401)	1,366 (367)	1,446 (377)
Sodium-to-Potassium Ratio	2.09 (0.94)	1.81 (0.72)	1.63 (0.58)	1.52 (0.52)
Cholesterol, mg/1000 kcal	170 (107.3)	181.7 (95.6)	182.4 (92.5)	188.7 (81.2)

*NDAM* Number of dishes in all meals

## Results

Tables 1 and 2 present the characteristics and nutritional status of the male and female participants according to NDAM. Participants in Group 4 (highest NDAM) were more likely to be older, non-smokers, and to report having an exercise habit, in both men and women. In contrast, both men and women in Group 1 (lowest NDAM) were more likely to be in the 20–49 age group, reside in the Kinki I area, and be current smokers. Regarding food group intake, participants in Group 4 had higher consumption of vegetables, fruits, fish, pulses, and milk, and lower consumption of cereals and meat. Regarding nutritional status, both men and women in Group 4 had a higher consumption of total energy, protein, polyunsaturated fatty acids, n-3 fatty acids, dietary fiber, and magnesium. They also had lower dietary energy densities and sodium/potassium ratios.

Figure 1 shows the multivariable-adjusted RRs and 95% CI for CVD risk factors according to NDAM in men. For men, compared with Group 1, the multivariable RRs of dyslipidemia were 0.869 (95% CI: 0.767–0.985) for Group 2, 0.932 (95% CI: 0.878–0.990) for Group 3, and 0.955 (95% CI: 0.919–0.999) for Group 4 (P for trend = 0.073). However, these associations did not reach statistical significance after the Bonferroni correction. The association with dyslipidemia was largely consistent in the sensitivity analysis, which included participants taking drugs other than lipid-lowering agents. Specifically, although the association was significant for Groups 2 and 3 before adjustment for multiple comparisons, this was not the case for Group 4 (Supplemental Table 1).

Figure 2 shows the multivariable-adjusted RRs and 95% CI for CVD risk factors according to NDAM in women. For women, the multivariable RR for overweight/obesity in Group 4 was 0.889 (95% CI: 0.792–0.999) compared with that in Group 1 (P for trend = 0.103). The multivariable RRs for hypertension compared with Group 1 were 0.712 (95% CI: 0.534–0.951) in Group 2, 0.843 (95% CI: 0.730–0.975) in Group 3, and 0.952 (95% CI: 0.858–1.063) in Group 4. However, these associations were not statistically significant after Bonferroni correction. The associations between overweight/obesity and hypertension were consistent in the sensitivity analysis, although some associations were not significant (Supplemental Table 2).

## Discussion

To our knowledge, this is the first study to investigate whether dietary diversity measured using the NDAM is associated with CVD risk factors in Japanese adults. Our findings suggest a potential protective effect of high-NDAM levels on several CVD risk factors. Specifically, in men, higher NDAM (Groups 2–4) was associated with a lower risk of dyslipidemia compared to Group 1 (lowest NDAM). In women, a similar inverse association was observed for overweight/obesity, with Group 4 (highest NDAM) showing a lower risk than Group 1. Furthermore, a lower risk of hypertension was found in Groups 2 and 3 compared with Group 1. However, these associations were not statistically significant after Bonferroni correction. Considering the potential for Type II errors associated with strict adjustments [17], we have discussed the results both before and after Bonferroni correction.

Based on our previous study [5], which found that higher NDAM was related to excessive cholesterol intake, we hypothesized that the RRs of dyslipidemia would increase with higher NDAM. However, although the cholesterol intake was higher among the higher NDAM groups, contrary to our expectations, the present study found that the RRs of dyslipidemia in men were lower. A possible explanation for this discrepancy may be other nutritional characteristics associated with higher NDAM, such as higher intake of dietary fiber and polyunsaturated fatty acids. Two meta-analyses of randomized controlled trials have shown that dietary fiber has a beneficial effect on serum lipids by lowering total cholesterol [18, 19] and LDL-C [19] Another meta-analysis showed that polyunsaturated fatty acid intake slightly decreases triglycerides [20]. These findings suggest that the beneficial effects of these nutrients in a high-NDAM diet may have outweighed the potential negative effects of higher cholesterol intake, thus contributing to lower RRs. In contrast, no clear preventive effect of higher NDAM levels was observed among women. One reason for this may be that, in the present study, NDAM was higher among women aged > 60 years, an age group that often experiences a postmenopausal increase in LDL-C [21]. This age-related increase in LDL-C may have masked the potential benefits of the diet. Notably, when the analysis was restricted to women in their 40s and younger (data not shown), the results were similar to those observed in men, although the association was not statistically significant.

The lower risk of overweight/obesity in women with the highest NDAM may be attributable to several nutritional characteristics of their diets, namely, lower dietary energy density and higher protein intake. A previous systematic review linked high dietary energy density to an increased risk of excess adiposity, weight gain, and higher BMI [22]. In contrast, low-energy-density diets, typically rich in vegetables and fruits, are associated with a low BMI or WC [15, 23] in the US [14] and Japan [24]. Furthermore, high protein intake can aid in weight management [25] through two mechanisms: enhancing satiety, which reduces total energy intake [26] and increasing diet-induced thermogenesis [27], which elevates total energy expenditure. In the sensitivity analysis, which included participants on medication, the RRs of overweight/obesity tended to decrease as NDAM increased, in both women and men.

Our previous study using the 2012 NHNS data showed that, in both men and women, higher NDAM was associated with greater sodium intake [5], a known risk factor for hypertension [6]. However, a preliminary analysis based on the same 2012 data revealed a protective effect against hypertension among women: groups with higher NDAM levels showed lower RRs of hypertension compared with the lowest NDAM group. This finding among women was consistent with our current 2018–2019 analysis. The reason for this protective effect against hypertension may be the inverse association between NDAM and the Na/K ratio, a known protective factor [28]. The inverse association was consistent in both the 2012 and 2018–2019 analyses, although the association between NDAM and sodium intake differed between 2012 and 2018–2019 among women. Thus, the consistently low Na/K ratio likely explains the lower RRs of hypertension observed among women with high NDAM. Among men, although less clear than in women and not statistically significant, the RR of hypertension was also lower in Group 2 compared with that in Group 1.

These results suggest that high-NDAM groups (Groups 2–4, i.e., eight or more dishes) may be useful for preventing hypertension in women. However, within these high-NDAM groups, as NDAM increased from Group 2 to Groups 3 and 4, the RRs of hypertension also tended to increase, though they remained lower than those in Group 1. This upward trend was also observed for the risk of overweight/obesity and abdominal obesity in women, and for dyslipidemia in men, which may be attributable to increased total energy intake with higher NDAM. Therefore, further research is needed to determine the most useful number of dishes for preventing hypertension and other CVD risk factors.

The strength of this study is that the data were collected from a nationally representative population, which reduced the risk of selection bias and enhanced the generalizability of our findings. However, this study has several limitations. First, certain types of meals in the NDAM, such as “one-plate dishes” and ‘*bento*’ (Japanese box lunches), may have been misclassified. Ideally, these multicomponent meals should be broken down into their constituent parts (e.g., grain dishes, egg dishes, and vegetable dishes). However, in the NHNS 2018–19 dataset, the data were recorded as single items. Consequently, in this study, we were unable to separate the components and classified them as one dish. To address this issue, we conducted a sensitivity analysis. Our dataset included 174 participants (6.0%) who reported consuming these types of meals, and when these participants were excluded, the results remained largely unchanged. Second, dietary intake was assessed using a one-day weighed dietary record, which may not reflect usual intake because of day-to-day variation in food consumption and could have resulted in misclassification of participants’ usual dietary patterns and NDAM. Finally, owing to the cross-sectional nature of the study, the possibility of reverse causation between NDAM and CVD risk factors cannot be ruled out. However, this possibility is likely mitigated by our exclusion of participants currently taking medications (anti-hypertensive, anti-hyperglycemic, cholesterol-lowering, and serum triglyceride-lowering) or those with a diagnosis of diabetes mellitus. This exclusion criterion helps to minimize the potential for reverse causality, whereby preexisting conditions influence dietary habits. Nevertheless, it is possible that some participants may have intentionally reduced their number of dishes for weight control.

## Conclusions

In conclusion, a diet high in NDAM may be inversely associated with several CVD risk factors among Japanese adults. However, longitudinal studies are needed to clarify the temporal relationship between NDAM and CVD risk factors and to identify the most effective number of dishes for CVD risk prevention.

## Supplementary Information

Supplementary Material 1.

## Data Availability

The data that support the findings of this study are available from the Ministry of Health, Labour and Welfare, Japan, but restrictions apply to the availability of these data, which were used under license for the current study, and so are not publicly available. Data are however available from the Ministry upon reasonable request and with appropriate official approval.

## Abbreviations

BMI: Body mass index
CI: Confidence intervals
CVD: Cardiovascular disease
DBP: Diastolic blood pressure
HDL-C: HDL cholesterol
LDL-C: LDL cholesterol
NDAM: Number of dishes in all meals
NHNS: The National Health and Nutrition Survey
Non-HDL-C: Non-HDL cholesterol
SBP: Systolic blood pressure
TG: Triglyceride
RR: Risk ratio
WC: Waist circumference
