# Supplementary material for: Association of dietary diversity measured by the number of dishes with cardiovascular risk factors among Japanese adults: findings from the National Health and Nutrition Survey, 2018-19
Source: Nutr J. 2026 May 12;25:71. doi: 10.1186/s12937-026-01326-6 (PMC13335204; doi:10.1186/s12937-026-01326-6)
Supplement: Supplementary file 1 — Supplementary Material 1. [file 12937_2026_1326_MOESM1_ESM.docx]

**Supplementary Table 1: Multivariate-adjusted RRs and 95% CIs for CVD risk factors according to the NDAM in men**

|  | Group 1 ≤ 7 dishes | Group 2 8-9 dishes | | | | | | Group 3 10-12 dishes | | | | | | Group 4 ≥ 13 dishes | | | | | | *P* for trend |
| --- | --- | --- | --- | --- | --- | --- | --- | --- | --- | --- | --- | --- | --- | --- | --- | --- | --- | --- | --- | --- |
| Overweight / obesity (n=2,158) ^a^ | n=499, 23.1% | n=410, 19.0% | | | | | | n=653, 30.3% | | | | | | n=596, 27.6% | | | | | |  |
| No. of cases (BMI ≥ 25) | 184 | 160 | | | | | | 228 | | | | | | 190 | | | | | |  |
| Multivariate model | 1.00 | 1.00 | ( | 0.84 | - | 1.19 | ) | 0.93 | ( | 0.86 | - | 1.02 | ) | 0.94 | ( | 0.88 | - | 1.00 | ) | 0.003 |
| Abdominal obesity (n=2,158) ^a^ |  |  |  |  |  |  |  |  |  |  |  |  |  |  |  |  |  |  |  |  |
| No. of cases (WC≥ 85cm) | 286 | 257 | | | | | | 408 | | | | | | 383 | | | | | |  |
| Multivariate model | 1.00 | 1.04 | ( | 0.93 | - | 1.16 | ) | 1.00 | ( | 0.95 | - | 1.06 | ) | 1.01 | ( | 0.97 | - | 1.05 | ) | 0.815 |
| Hypertension　(n=1,383) ^b^ | n=366, 26.4% | n=277, 20.0% | | | | | | n=373, 27.0% | | | | | | n=367, 26.5% | | | | | |  |
| No. of cases (SBP≥ 140mmHg, DBP≥ 90mmHg) | 117 | 94 | | | | | | 138 | | | | | | 123 | | | | | |  |
| Multivariate model | 1.00 | 0.91 | ( | 0.73 | - | 1.14 | ) | 0.98 | ( | 0.88 | - | 1.10 | ) | 0.96 | ( | 0.88 | - | 1.04 | ) | 0.149 |
| Diabetes Mellitus (n=1,706) ^c^ | n=409, 24.0% | n=321, 18.8% | | | | | | n=517, 30.3% | | | | | | n=459, 26.9% | | | | | |  |
| No. of cases (HbA1c≥6.5 %) | 13 | 5 | | | | | | 27 | | | | | | 19 | | | | | |  |
| Multivariate model | 1.00 | 0.72 | ( | 0.22 | - | 2.38 | ) | 1.11 | ( | 0.81 | - | 1.51 | ) | 1.15 | ( | 0.84 | - | 1.58 | ) | 0.563 |
| Dyslipidemia (n=1,777) ^d^ | n=439, 24.7% | n=336, 18.9% | | | | | | n=533, 30.0% | | | | | | n=469, 26.4% | | | | | |  |
| No. of cases (HDL-C<40 mg/dL, LDL-C≥120 mg/dL, TG≥150 mg/dL, nonHDL-C≥150 mg/dL) | 305 | 210 | | | | | | 323 | | | | | | 310 | | | | | |  |
| Multivariate model | 1.00 | 0.88 | ( | 0.79 | - | 0.98 | ) | 0.93 | ( | 0.88 | - | 0.98 | ) | 0.97 | ( | 0.93 | - | 1.00 | ) | 0.267 |

RR: risk ratios, CI: confidence intervals, CVD: cardiovascular disease, NDAM: number of dishes in all meals, BMI: body mass index, WC: waist circumference, ﻿SBP: systolic blood pressure, DBP: diastolic blood pressure, HbA1c: hemoglobin A1c, HDL-C: HDL-cholesterol, LDL-C: LDL-cholesterol, TG: triglyceride, non-HDL-C: non-HDL-cholesterol.

^a^ Including participants with current medication (anti-hypertensive, anti-hyperglycemic, cholesterol-lowering, serum triglyceride–lowering) and participants with diagnosis of diabetes mellitus.

^b^ Excluding participants with current medication (anti-hypertensive)

^c^ Excluding participants with diagnosis of diabetes mellitus

^d^ Excluding participants with current medication (anti-hyperglycemic, cholesterol-lowering, serum triglyceride–lowering)

Analyzed by modified Poisson regression model.

Multivariable model: adjusted for age, living alone, area, occupation, smoking status, drinking status, exercise habits, and total energy intake.

*P* for trend is calculated across the median values of NDAM.

**Supplementary Table 2: Multivariate-adjusted RRs and 95% CIs for CVD risk factors according to the NDAM in women**

|  | Group 1 ≤ 7 dishes | Group 2 8-9 dishes | | | | | | Group 3 10-12 dishes | | | | | | Group 4 ≥ 13 dishes | | | | | | *P* for trend |
| --- | --- | --- | --- | --- | --- | --- | --- | --- | --- | --- | --- | --- | --- | --- | --- | --- | --- | --- | --- | --- |
| Overweight / obesity (n=2,990) ^a^ | n=557, 18.6% | n=619, 20.7% | | | | | | n=976, 32.6% | | | | | | n=838, 28.0% | | | | | |  |
| No. of cases (BMI ≥ 25) | 142 | 140 | | | | | | 239 | | | | | | 191 | | | | | |  |
| Multivariate model | 1.00 | 0.79 | ( | 0.64 | - | 0.98 | ) | 0.90 | ( | 0.82 | - | 1.00 | ) | 0.88 | ( | 0.82 | - | 0.95 | ) | 0.003 |
| Abdominal obesity |  |  |  |  |  |  |  |  |  |  |  |  |  |  |  |  |  |  |  |  |
| No. of cases (WC≥ 90cm) ^a^ | 121 | 135 | | | | | | 229 | | | | | | 195 | | | | | |  |
| Multivariate model | 1.00 | 0.86 | ( | 0.69 | - | 1.08 | ) | 0.93 | ( | 0.84 | - | 1.03 | ) | 0.93 | ( | 0.86 | - | 1.00 | ) | 0.031 |
| Hypertension (n=2,202) ^b^ | n=443, 20.1% | n=469, 21.3% | | | | | | n=700, 31.7% | | | | | | n=590, 26.8% | | | | | |  |
| No. of cases (SBP≥ 140mmHg, DBP≥ 90mmHg) | 91 | 102 | | | | | | 156 | | | | | | 145 | | | | | |  |
| Multivariate model | 1.00 | 0.79 | ( | 0.62 | - | 1.02 | ) | 0.87 | ( | 0.77 | - | 0.98 | ) | 0.96 | ( | 0.87 | - | 1.05 | ) | 0.083 |
| Diabetes Mellitus (n=2,634) ^c^ | n=511, 19.4% | n=557, 21.1% | | | | | | n=828, 31.4% | | | | | | n=738, 28.0% | | | | | |  |
| No. of cases (HbA1c≥6.5 %) | 10 | 16 | | | | | | 14 | | | | | | 22 | | | | | |  |
| Multivariate model | 1.00 | 1.18 | ( | 0.50 | - | 2.80 | ) | 0.69 | ( | 0.44 | - | 1.08 | ) | 0.96 | ( | 0.70 | - | 1.31 | ) | 0.620 |
| Dyslipidemia (n=2,315) ^d^ | n=473, 20.4% | n=487, 21.0% | | | | | | n=750, 32.4% | | | | | | n=605, 26.1% | | | | | |  |
| No. of cases (HDL-C<40 mg/dL, LDL-C≥120 mg/dL, TG≥150 mg/dL, non-HDL-C≥150 mg/dL) | 276 | 300 | | | | | | 465 | | | | | | 390 | | | | | |  |
| Multivariate model | 1.00 | 1.01 | ( | 0.91 | - | 1.12 | ) | 0.98 | ( | 0.93 | - | 1.03 | ) | 1.00 | ( | 0.96 | - | 1.04 | ) | 0.818 |

RR: risk ratios, CI: confidence intervals, CVD: cardiovascular disease, NDAM: number of dishes in all meals, BMI: body mass index, WC: waist circumference, ﻿SBP: systolic blood pressure, DBP: diastolic blood pressure, HbA1c: hemoglobin A1c, HDL-C: HDL-cholesterol, LDL-C: LDL-cholesterol, TG: triglyceride, non-HDL-C: non-HDL-cholesterol.

^a^ Including participants with current medication (anti-hypertensive, anti-hyperglycemic, cholesterol-lowering, serum triglyceride–lowering) and participants with diagnosis of diabetes mellitus.

^b^ Excluding participants with current medication (anti-hypertensive)

^c^ Excluding participants with diagnosis of diabetes mellitus

^d^ Excluding participants with current medication (anti-hyperglycemic, cholesterol-lowering, serum triglyceride–lowering)

Analyzed by modified Poisson regression model.

Multivariable model: adjusted for age, living alone, area, occupation, smoking status, drinking status, exercise habits, and total energy intake.

*P* for trend is calculated across the median values of NDAM.
